# Targeting the selectivity filter to drastically alter the activity and substrate spectrum of a promiscuous metal transporter

**DOI:** 10.1039/d5sc03700j

**Published:** 2025-08-26

**Authors:** Yuhan Jiang, Michael Nikolovski, Tianqi Wang, Keith MacRenaris, Thomas V. O'Halloran, Jian Hu

**Affiliations:** a Department of Chemistry, Michigan State University East Lansing Michigan USA ohallor8@msu.edu hujian1@msu.edu; b Department of Biochemistry and Molecular Biology, Michigan State University East Lansing Michigan USA; c Department of Microbiology, Genetics, & Immunology, Michigan State University East Lansing Michigan USA; d Elemental Health Institute, Michigan State University East Lansing Michigan USA; e Quantitative Bio Element Analysis and Mapping (QBEAM) Center, Michigan State University East Lansing Michigan USA

## Abstract

d-Block metal transporters are attractive engineering targets for selectively enriching or excluding metals in living organisms. However, systematic efforts to engineer these transporters have been hindered by limited knowledge on the determinants of substrate specificity. Here, we applied a focused-screen approach to human ZIP8, a promiscuous d-block divalent metal transporter, by systematically changing the residues that form a proposed selectivity filter at the entrance of the transport pathway. Library screening using a cell-based transport assay quantified by ICP-MS led to the identification of the variants with drastically altered transport activities and substrate preferences, including a variant that exhibits new activities for the non-substrate metals VO^2+^ and Cu^2+^. Together with the identification of Pb^2+^ as a new substrate of ZIP8, these findings indicate that the ZIP fold is highly adaptable and amenable for transporting a wide range of metals, making it a promising scaffold to generate novel metal transporters for applications.

## Abstract 2

*d*-Block metal transporters are attractive engineering targets for selectively enriching or excluding metals in living organisms.

## Graphical abstract

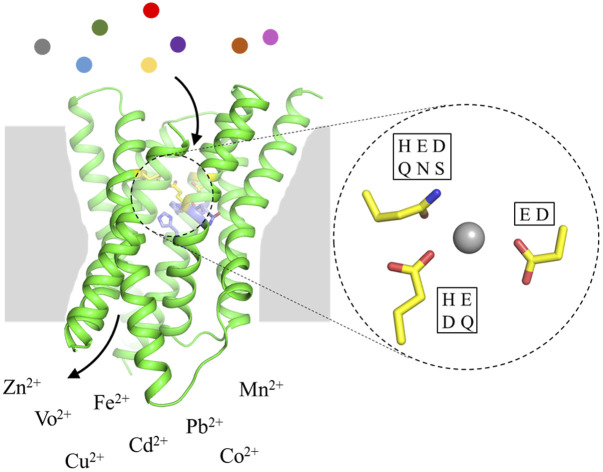

## Introduction

The d-block metals manganese, iron, cobalt, nickel, copper, zinc, and molybdenum perform essential functions in biological systems by playing structural, catalytic, and regulatory roles in various biomolecules.^1–7^ The numerous physiological functions of these metals and the potential deleterious effects upon dysregulation underscore the need for precise control over their concentrations and distributions within living organisms. Metal transporters, which function as selective gates to regulate the flow of metal ions across biological membranes, are central players in maintaining the homeostasis of these micronutrients at cellular and systemic levels.^8,9^

The divalent d-block metal transporters are considered to be attractive engineering targets for applications such as biofortification,^10,11^ phytoremediation,^12,13^ biomining,^14,15^ and heavy metal exclusion from food.^16,17^ From this perspective, the Zrt-/Irt-like protein (ZIP) family has gained increasing attention due to its ubiquitous expression in all kingdoms of life, its broad substrate spectrum and, in particular, its pivotal role in metal uptake from the environment.^18–22^ Creating ZIPs with desired properties will allow a highly selective accumulation or exclusion of specific metals in the host organism. For example, engineering IRT1, a plant root-expressing iron-transporting ZIP, to eliminate Cd transport activity while retaining Fe transport activity may help reduce Cd uptake by crops grown on the contaminated lands.^23^

**Fig. 1:**
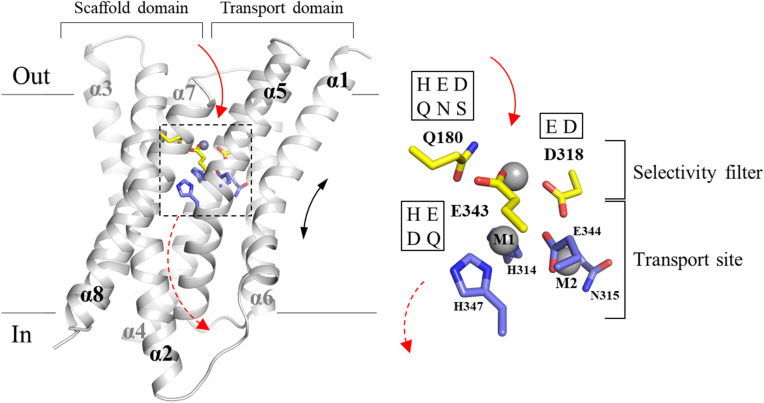
The selectivity filter and transport site of ZIP8. Left: the structural model of human ZIP8 in the outward-facing conformation. The solid arrow indicates the pathway for metal to enter the transport site through the selectivity filter, and the dashed arrow indicates the pathway for metal to be released to the cytoplasm when the transporter switches to the inward-facing conformation. The black double-headed arrow indicates the elevator motion of the transport domain (transmembrane helix 1/4/5/6) relative to the scaffold domain (transmembrane helix 2/3/7/8). The model was generated by homology modeling using the AlphaFold predicted ZIP13 structure (in an outward-facing conformation) as template (UniProt ID: Q96H72). For clarity, the long loops connecting transmembrane helices are trimmed. The residues at the selectivity filter are shown in yellow and the residues forming the transport site are in blue. Note that D318 and E343 contribute to both the selectivity filter and the transport site. The metal substrate modeled at the selectivity filter is depicted as a grey sphere. Right: the zoomed-in view of the residues in the selectivity filter and transport site. Metals that are hypothetically bound at the transport site (M1 and M2) and the selectivity filter are depicted as grey spheres. The one-letter codes of the amino acids tested at the indicated positions in this work are shown in the frames.

ZIP8 is a promiscuous ZIP that transports multiple d-block divalent metal ions (Zn^2+^, Fe^2+^, Mn^2+^, Co^2+^, and Cd^2+^) and crucially contributes to manganese homeostasis in humans.^24–30^ In our previous study, we showed that a combination of four mutations led to a variant with increased Zn^2+^ transport activity and drastically reduced activities for Cd^2+^, Fe^2+^, and Mn^2+^, demonstrating the feasibility of rational engineering of a ZIP transporter.^31^ The strong epistatic interaction between the two residues selected for mutagenesis led to the identification of a selectivity filter located at the entrance of the transport pathway (Fig. 1). According to the proposed elevator transport mode,^32–34^ E318 and E343 from the transport domain approach Q180 in the scaffold domain when the transporter adopts an outward-facing conformation, thus forming a transient metal binding site that screens incoming metal ions before they reach the transport site. Bioinformatics analysis of these positions showed that, although the selectivity filter-like structure is likely present in many ZIPs, the amino acid composition is highly variable,^31,35^ suggesting that these transporters may have different substrate preferences, and that the selectivity filter represents an ideal target to alter the substrate spectra of ZIPs.

In this work, we explored the extent to which the transport properties of ZIP8 can be tuned by systematically altering the amino acid composition of the selectivity filter and screening the variants against a panel of d-block metals in an established cell-based assay using inductively coupled plasma mass spectrometry (ICP-MS) for metal quantification.^36^ This approach allowed us to identify the variants with drastically altered transport activities and substrate preferences, demonstrating the great potential of the ZIP fold for selective metal transport in transporter engineering.

## Results

### Construction of a ZIP8 variant library

Since the selectivity filter is at the forefront of metal interaction, we hypothesized that changing the amino acid composition of the selectivity filter would significantly change substrate preference. To test this, we systematically introduced polar amino acid residues at these positions based on the bioinformatics analysis of the ZIP family (Fig. 1).^35^ Q180 is at the pore entrance and this position is mostly occupied by a polar or charged residue, including histidine, aspartate, glutamate, asparagine, glutamine, and serine.^31^ E318 is one of the residues at the transport site and part of the selectivity filter. In other ZIPs, this position can also be occupied by aspartate or histidine.^35^ A histidine at this position is present only in ZIP13, an ER/Golgi-residing ZIP that was shown to be a zinc importer^37^ but then identified as an iron exporter, *i.e.* transporting iron from the cytoplasm to the lumen of ER/Golgi-complex.^38–40^ In this work, histidine was not introduced to this position because the corresponding selectivity filter-like structure in ZIP13 is unlikely to screen metals if ZIP13 maintains the same topology as other ZIPs but functions as an exporter. Instead, this histidine in ZIP13 likely plays a role at the exit of the transport pathway and thus should not be viewed and tested as a residue in the selectivity filter. E343 is another residue that plays roles in both the selectivity filter and the transport site. In addition to glutamate, the E343 position can also be occupied by histidine or glutamine in other ZIPs.^31^ Given the similar property to glutamate, aspartate was also introduced to this position although it is not present at this position in any known ZIP. Eventually, a library consisting of 48 constructs (wild-type ZIP8 and 47 variants) was generated for functional screen (Table 1).

**Table 1:** Screening of the ZIP8 construct library against mixtures of metal substrates

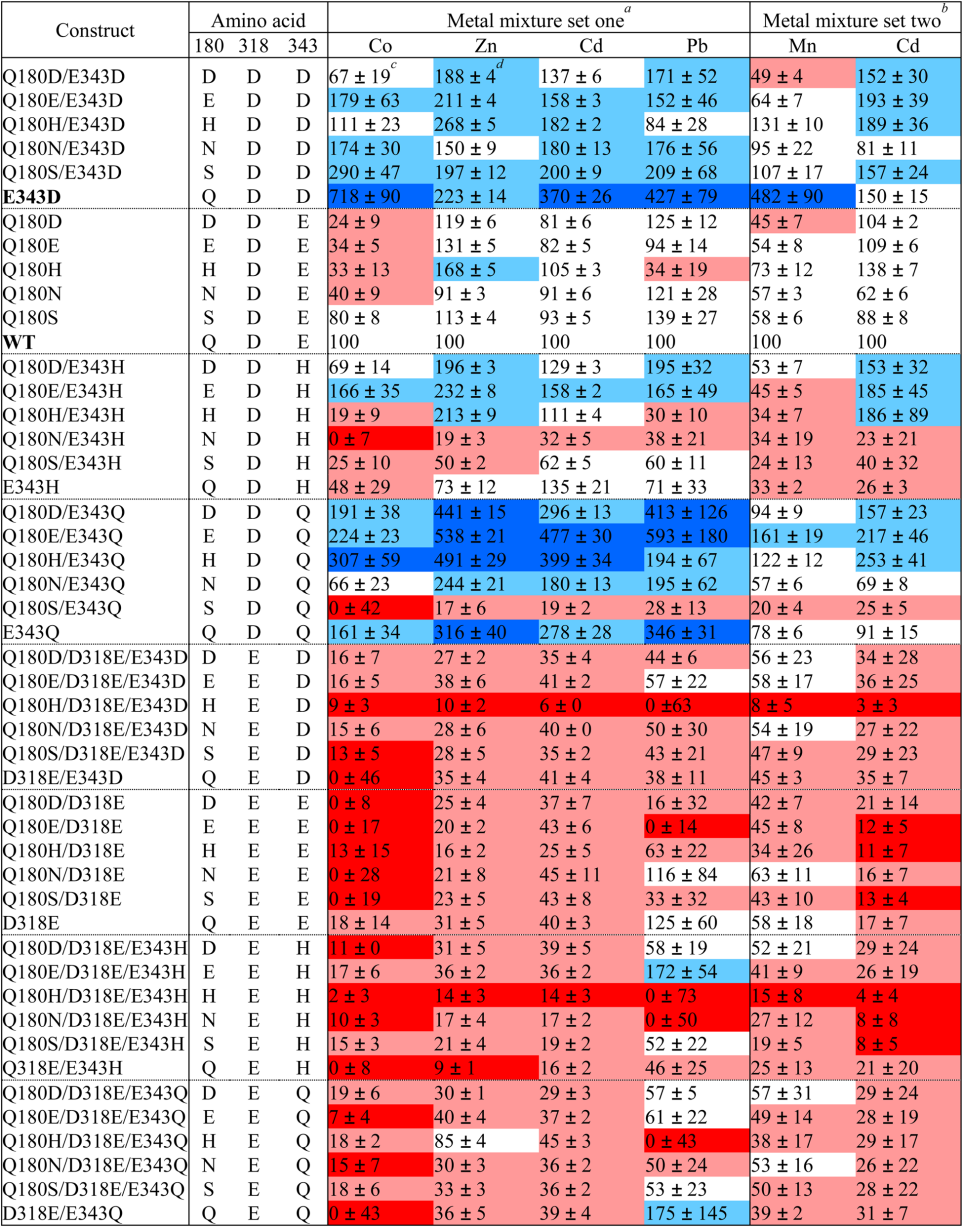

a Composed of 5 μM CdCl_2_, CoCl_2_, MnCl_2_, Pb(NO_3_)_2_, ^57^FeCl_3_, and ^70^ZnCl_2_.

b Composed of 2 μM of CdCl_2_, 15 μM of MnCl_2_, 40 μM of ^57^FeCl_3_, and 1 mM of ascorbic acid. Fe^2+^ data are not shown due to low activity and large variation.

c Mean ± S.E. Activity is expressed as a percentage of the activity of wild-type ZIP8. The numbers of independent experiments for set one and set two are four and three, respectively.

d Cells are colored as follows: less than 15%, red; between 15% and 50%, pink; between 50% and 150%, white; between 150% and 300%, light blue; greater than 300%, dark blue.

### Metal screening and identification of Pb^2+^ as a new substrate of ZIP8

**Fig. 2:**
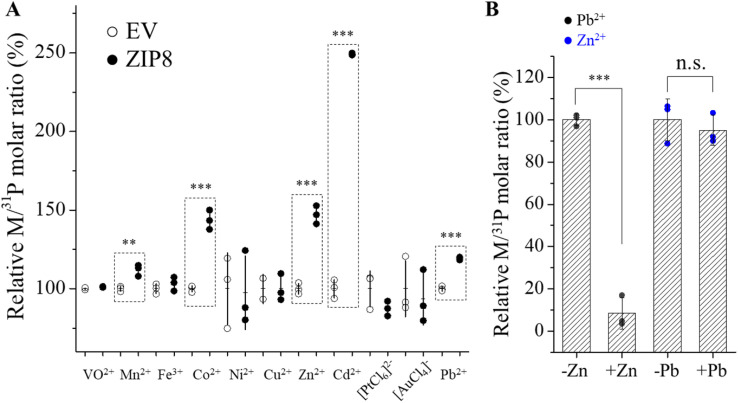
Detection of ZIP8 transport activities for a panel of metal ions. (A) Screening for cellular metal content after 30 min of uptake using ICP-MS. Cells were incubated in a buffer containing a mixture of indicated metals. The relative M/^31^P molar ratio of the ZIP8 group is expressed as the percentage of the M/^31^P molar ratio of the empty vector group. Each symbol represents the result of one of three replicates for each condition. The horizontal bar is the mean of three replicates and the vertical bar shows 1 ± S.D. Two-tailed Student's *t*-test was performed to examine statistical significance. **: *P* < 0.01; ***: *P* < 0.001. The *P* values for Mn^2+^, Co^2+^, Zn^2+^, Cd^2+^, and Pb^2+^ are 0.006, 0.0003, 0.0003, 2 × 10^−6^, and 4 × 10^−5^, respectively. (B) Transport of Pb^2+^ (or Zn^2+^) at 5 μM in the absence and presence of Zn^2+^ (or Pb^2+^) at 50 μM. The shown data are from one of two independent experiments with similar results.

In order to define the border of the substrate spectrum of wild-type ZIP8, we screened a mixture containing a total of eleven metals at the same concentration of 5 μM for each metal, including the known ZIP8 substrates (Zn as ^70^Zn, Fe as ^57^Fe, Mn, Co, and Cd) and those that are not (V as VO^2+^, Ni, Cu, Pb, Au as [AuCl_4_]^−^, and Pt as [PtCl_6_]^2−^), in the presence of 2 mM CaCl_2_ and 1 mM MgCl_2_. Different from our previous studies where a Chelex-treated cell culture medium (DMEM plus 10% fetal bovine serum) was used in the transport assay, we used a chelator-free, serum-free buffer in this work to avoid differential metal binding to serum proteins and small molecule chelators in the cell culture medium. In the transport assay, HEK293T cells transiently expressing ZIP8 (or its variants) were incubated with the buffer containing the metal mixture, and ICP-MS was used to measure the content of each metal in cells, which are expressed as the molar ratio of metal and phosphorus (M/^31^P). Our previous study on ZIP4 showed that using phosphorus to calibrate metal uptake improved data precision by levelling out variations in cell number between samples.^36^ As expected, the uptake of the known ZIP8 substrates, including Zn^2+^, Cd^2+^, Mn^2+^ and Co^2+^, into the cells expressing ZIP8 were significantly higher than the cells transfected with an empty vector (Fig. 2A), indicative of the transport activities for these metals. No transport activity of ^57^Fe, a stable Fe isotope with a low natural abundance (2.1%), was detected because ascorbic acid, a reducing agent that would reduce Fe^3+^ to Fe^2+^, is incompatible with several metals (Cu^2+^, Pb^2+^, VO^2+^, [AuCl_4_]^−^, and [PtCl_6_]^2−^) and was therefore excluded from the metal mixture. The lack of ^57^Fe^3+^ transport activity is consistent with the notion that Fe^3+^ is not a substrate of ZIP8, an established Fe^2+^ transporter.^26^ Unexpectedly, the transport activity for Pb^2+^ was detected as the Pb/^31^P ratio for the cells expressing ZIP8 was significantly higher than that of the cells transfected with an empty vector (Fig. 2A). In the metal competition assay, 10-fold excess Zn^2+^ almost abolished Pb^2+^ uptake, but 10-fold excess Pb^2+^ did not significantly reduce Zn^2+^ transport (Fig. 2B), suggesting that the affinity for Pb^2+^ is much lower than that for Zn. It has been shown that only very high concentration of Pb^2+^ at a 30-fold excess was able to significantly block ZIP8-mediated Zn^2+^ and Cd^2+^ transport.^27^ The low affinity for Pb^2+^ was then supported by a kinetic study shown in a later section. As the radius of Pb^2+^ (119 pm) is much larger than Zn^2+^ (74 pm), identification of Pb^2+^ as a substrate indicates that the transport pathway of ZIP8 is flexible enough to allow the transport of metal ions with diverse sizes. Even so, the inability of wild-type ZIP8 to transport VO^2+^, Ni^2+^, Cu^2+^, [AuCl_4_]^−^, and [PtCl_6_]^2−^ indicates that despite its promiscuity, ZIP8 still transports metals in a selective manner.

### Library screening to identify variants with altered transport properties

**Fig. 3:**
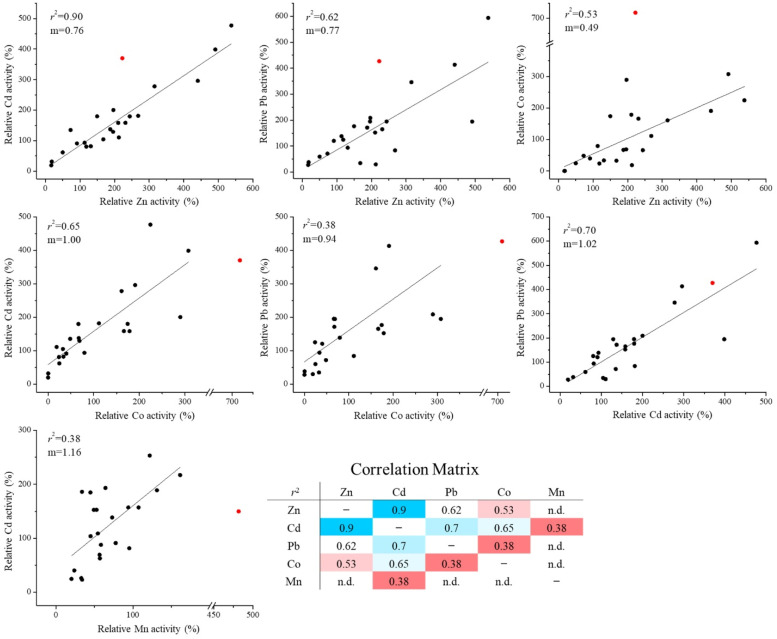
Correlation analysis of the activities of different metal substrates of 24 constructs of ZIP8. Only the variants without the D318E substitution were analyzed due to the strong detrimental effects of the D318E mutation on the activities for nearly all metals tested. Activities are expressed as percentages of the corresponding activities of wild-type ZIP8. The E343D variant (red sphere) was excluded from linear regression because it appears to be an outlier in several cases. The activities for Zn^2+^, Cd^2+^, Pb^2+^, and Co^2+^ obtained in metal mixture set one and the activities for Mn^2+^ and Cd^2+^ obtained in metal mixture set two were applied to correlation analysis. The *r*^2^ values are summarized in the correlation matrix table.

Since ICP-MS can quantify multiple elements in a single sample, it is particularly useful for studying substrate specificity of metal transporters, as the relative transport rate is not affected by the variations in expression of the transporter of interest among different samples. In this work, the 48 constructs in the library were screened against a mixture of metal substrates – Zn^2+^, Cd^2+^, Mn^2+^, Co^2+^, Fe^2+^, and Pb^2+^ (set one). However, due to the competition between the substrates, the activities of Mn^2+^ and Fe^2+^ couldn't be consistently measured, suggestive of their relatively low transport activities when compared to other metals. To address this problem, a second metal mixture composed of Mn^2+^, Fe^2+^, and Cd^2+^ (set two) was set up to screen the library. The molar ratios of Mn^2+^, Fe^2+^, and Cd^2+^ have been optimized so that their activities can be detected for wild-type ZIP8 (Fig. S1). The M/^31^P ratios of the variants were calculated and expressed as a percentage of the M/^31^P ratio for wild-type ZIP8, and the results from 3–4 independent experiments are summarized in Table 1. The expression of ZIP8 and its variants was detected by western blot (Fig. S2). The Fe^2+^ uptake data are not listed in Table 1 because the readings for many variants were less reproducible with larger deviations than those for other metals, likely due to the weak Fe^2+^ transport activity even under the optimized conditions. Kinetic studies of Fe^2+^ transport of wild-type ZIP8 and a selected variant (E343D) are described in the next section. Analysis of the data in Table 1 revealed the following findings:

(i) The D318E mutation is detrimental to the transport of nearly all metals. For most D318E-containing variants, the abolished activity was not due to altered expression according to the results of the western blot experiments (Fig. S2).

(ii) The mutations on E343 have different impacts on transport activity. The E343D mutation alone or in combination with Q180 mutations led to increased activities for most metals. Similarly, the E343Q mutation alone or in combination with Q180 mutations (except for Q180S) favored the transport of most metals. In contrast, the variants containing the E343H mutation exhibited very different activities. Among them, the double variant Q180H/E343H showed a selectively increased activity for Zn^2+^, consistent with our previous study.^31^ Q180H alone also improved selectivity for Zn^2+^, and the effect was strengthened when combined with E343H, as reported previously.^31^

(iii) There are positive correlations between the activities of any two metals (Fig. 3), when the E343D variant and the variants containing the D318E mutation are excluded. The strongest correlation was found between Zn^2+^ and Cd^2+^, which is consistent with their similar chemical properties and therefore it would be most challenging to create variants to selectively transport one while excluding the other. In contrast, the poorest correlations between Mn^2+^ and Cd^2+^ and between Co^2+^ and Pb^2+^ imply better chances to separate the activities for each metal pair. While the slopes of the Zn-involved linear regression were all smaller than one (Fig. 3, first row), indicating that most of the mutations at the selectivity filter led to an increased preference for Zn^2+^ over other metals, the E343D variant is an apparent outlier because it shows a preference for non-zinc metals.

### Kinetic study of the E343D variant

**Fig. 4:**
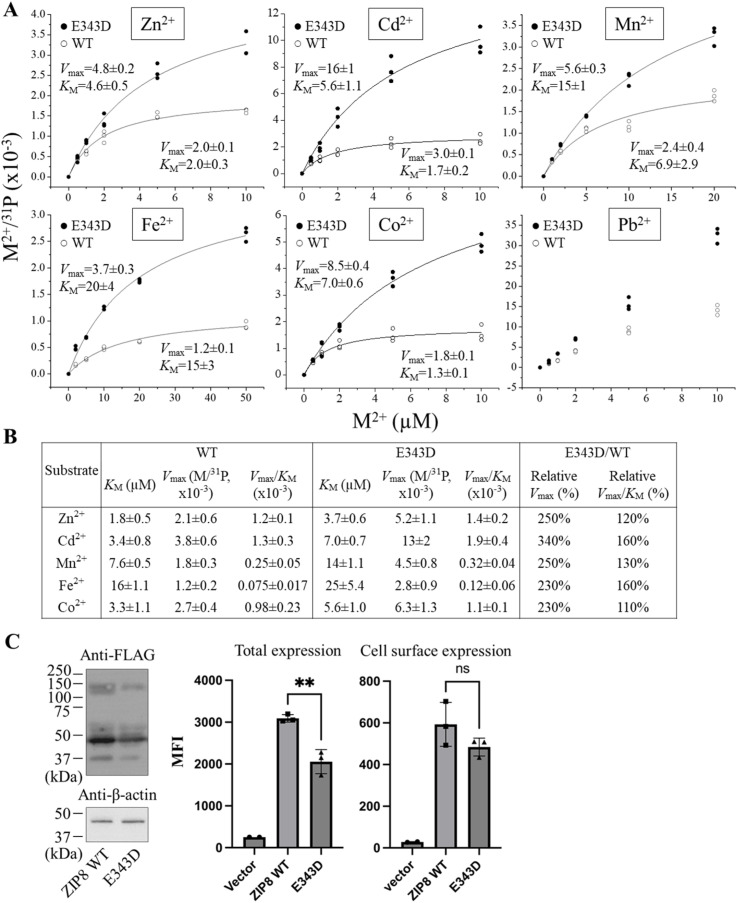
Kinetic study of the E343D variant in comparison with wild-type ZIP8. (A) Transport kinetics of six metal substrates. The M/^31^P molar ratios were plotted against the indicated metal concentrations. The shown data are from one of the three independent experiments with similar results. Curve fittings were conducted using the Michaelis–Menten model except for the curves for Co^2+^ and Pb^2+^. Each data point represents the result of one of the three biological replicates for one condition. The uncertainties shown in the figure are the standard errors of curve fitting. (B) Summary of the kinetic parameters from three independent experiments. *V*_max_ and *V*_max_/*K*_M_ are expressed as the percentage relative to wild-type ZIP8 measured in the same experiment. The data are expressed as mean ± S.E. (*n* = 3). (C) Comparison of the expression levels of wild-type ZIP8 and the E343D variant. Left: total expression of the FLAG-tagged ZIP8 and E343D variant detected by western blot. The total expression level of the variant is about half of that of wild-type ZIP8. Right: total expression and cell surface expression measured by flow cytometry using an anti-FLAG antibody and an Alexa Fluor 568-labeled anti-mouse antibody. MFI: mean fluorescence intensity. Cells were fixed with and without permeabilization prior to staining for detection of the total and cell surface expression, respectively. Two-tailed Student's *t*-test was used to examine statistical significance. **: *P* < 0.01. Data processing is illustrated in Fig. S4.

As the E343D variant exhibited transport properties different from the other variants, we performed kinetic studies to understand the mechanism of its broadly increased activities and altered substrate preference observed in the metal screen (Table 1). Kinetic studies were performed on single metals to avoid interference between competing substrates. The molar ratios of M/^31^P obtained from ICP-MS were plotted against the indicated metal concentrations, following by curve fitting using the Michaelis–Menten model. As shown in Fig. 4A and B, the results of the kinetic study consistently showed that the E343D variant exhibited a significant increase in *V*_max_ for Zn^2+^, Cd^2+^, Mn^2+^, and Fe^2+^ by 2–3 folds, while the *K*_M_ values for these metals were also increased. Although the E343D mutation did not significantly change the order of the *V*_max_/*K*_M_ values, *i.e.* Zn^2+^–Cd^2+^–Co^2+^ > Mn^2+^ > Fe^2+^, the variant showed enhanced transport of Cd^2+^ and Fe^2+^ more than the other metals. We noticed that the largely preferred transport for Co^2+^ and Mn^2+^ of this variant, as indicated in the screening of metal mixtures (Table 1 & Fig. 3), cannot be explained by the measured kinetic parameters. This discrepancy suggests that the metal substrates do not simply compete for the single high-affinity transport site, and therefore the assumption that the relative transport rate obtained from the experiments using a substrate mixture accurately reflects the ratios of the *V*_max_/*K*_M_ values determined using individual substrates does not hold any more. Indeed, the involvement of the selectivity filter, a weak but critical metal binding site, may complicate the kinetic analysis. Allostery between Zn^2+^ and Mn^2+^ was also noticed in our previous study on a ZIP8 variant.^31^ For Pb^2+^, we were not able to obtain the steady-state kinetic parameters: the dose–response curves were almost linear, suggestive of poor binding and consistent with the results of the competition experiment (Fig. 2B). Even so, it is clear that the E343D variant transports Pb^2+^ faster than the wild-type transporter. The increased transport activity was not due to a higher expression level of the variant. Western blot and flow cytometry data showed that the E343D variant actually has a lower total expression level but a similar cell surface expression level when compared to wild-type ZIP8 (Fig. 4C & S4). Therefore, the *k*_cat_ value of this variant is approximately 2–3 times higher than the wild-type transporter, as indicated by the relative *V*_max_ values (Fig. 4B). Taken together, the kinetic studies showed that the E343D variant transports all the metals tested more efficiently, with Cd^2+^ and Fe^2+^ being the most enhanced, while the apparent affinities for metals were all reduced.

### New transport activities for non-substrate metals

**Fig. 5:**
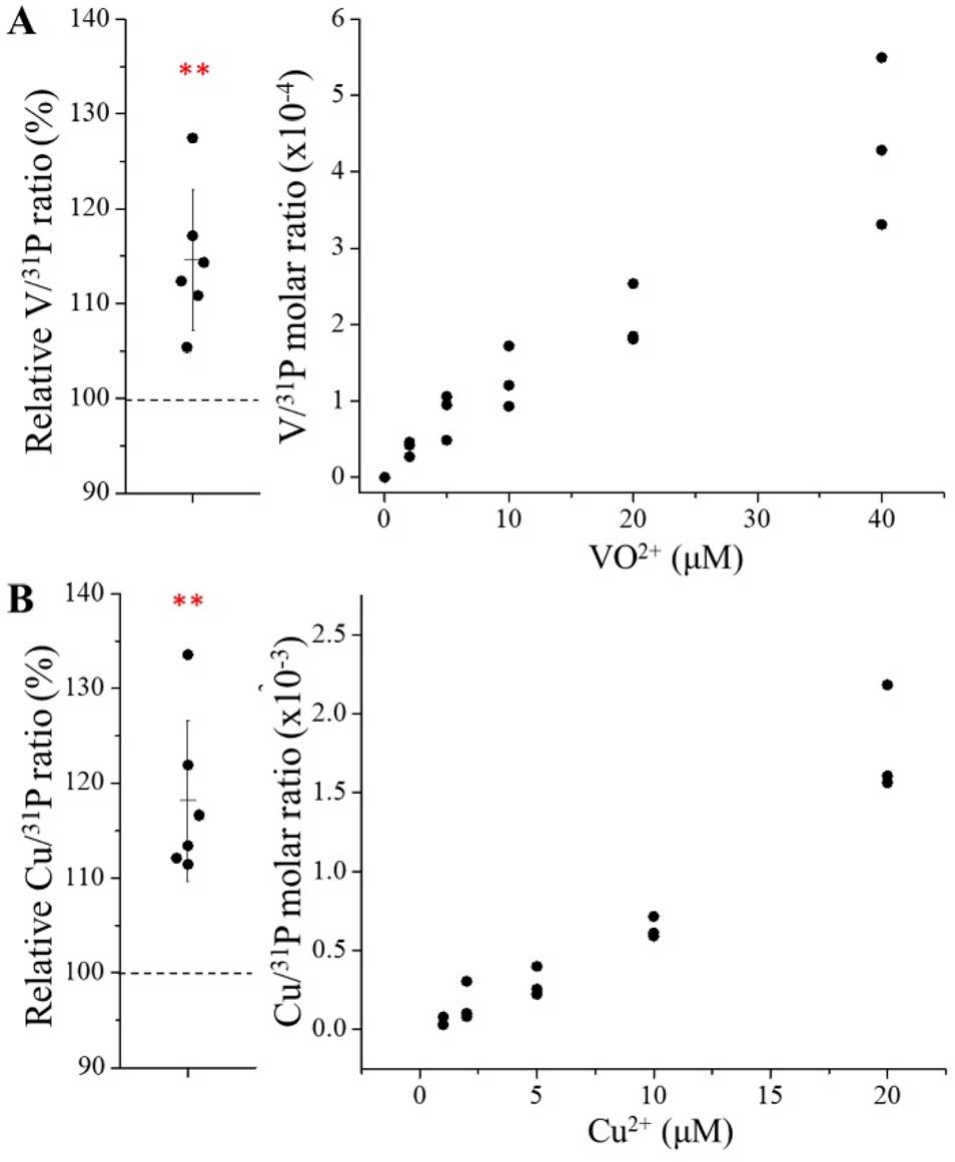
New transport activities of the E343D variant for VO^2+^ (A) and Cu^2+^ (B). The results of six independent experiments with cells incubated with 5 μM M^2+^ are shown on the left. The dashed lines indicate the normalized M^2+^ uptake by the cells transfected with an empty vector (set as 100%). Statistical analyses were performed using two-tailed Student's *t*-test. The *P* values for VO^2+^ and Cu^2+^ are 0.0048 and 0.0033, respectively. **: *P* < 0.01. The dose–response curves for VO^2+^ and Cu^2+^ from one of two independent experiments with similar results are shown on the right with three biological replicates tested for each condition.

Since the E343D variant exhibited a significantly increased transport efficiency and an altered substrate preference, we wondered if this variant could transport metals that are not the substrates for wild-type ZIP8. We then tested two divalent ions, VO^2+^, which has approximately twice hydrated radius of the known ZIP8 substrates, and Cu^2+^, which has been reported to be transported by various ZIPs^41,42^ but not by ZIP8 in this study (Fig. 2). Of great interest, the E343D variant exhibited transport activities for both metals (Fig. 5), even though the activities are marginal and the dose–response curves are not saturable, suggestive of weak binding. These results indicate that the E343D variant has an expanded substrate spectrum and thus becomes more promiscuous than wild-type ZIP8.

## Discussion

Engineering of d-block metal transporters for enrichment or exclusion of specific metals from living organisms has great potential for a variety of applications. For example, expressing importers specific for beneficial metals in crop roots may help to address mineral deficiency problems in populations,^10,11^ while carefully tuning the substrate spectra of these metal importers may provide a solution to avoid heavy metal accumulation in crops.^16,17^ In engineered plants/algae, overexpression of toxic metal-specific importers can remove heavy metals from the environment,^12,13^ and the same strategy can also be used to harvest low abundance precious metals from surroundings.^14,15^ However, these prospects still face several significant obstacles. First, the d-block metals that are the primary targets in these applications have similar physicochemical properties, making it challenging to create metal transporters with high selectivity. Second, our understanding of the transport mechanism and substrate specificity of d-block metal transporters is still in its infancy, and the poor knowledge of the dynamic interactions between d-block metals and transporters during metal translocation hinders rational engineering. Third, the lack of high-throughput assays for metal transporters impedes the application of directed evolution to metal transporter engineering. In this work, we focused on the selectivity filter at the pore entrance of ZIP8,^31^ a promiscuous d-block divalent cation transporter, to explore the potential of changing the transport properties, especially the substrate preference, for large-scale metal transporter engineering. Our results indicated that both the transport activity and substrate specificity of ZIP8 can be altered over a wide range (Table 1, Fig. 4 and 5), demonstrating the feasibility of tuning the function of a metal transporter by targeting residues selected based on prior knowledge of its structure and mechanism.

By mutating the three residues that form the selectivity filter, we generated a library consisting of 48 constructs and screened them using ICP-MS against metal mixtures. Among the variants, the E343D variant is particularly interesting because of the significantly enhanced transport activity and altered substrate preference. The kinetic study showed that the E343D mutation increased the transport rate (*V*_max_) at the cost of affinity (Fig. 4), which is similar to the affinity-activity trade-off for enzymes.^43^ Since the increase in *V*_max_ is greater than that in the apparent *K*_M_, the E343D variant exhibited enhanced transport efficiencies (*V*_max_/*K*_M_) for all tested metals with the transport efficiency of Cd^2+^ and Fe^2+^ increasing most. Structurally, as E343 participates in both the selectivity filter and the transport site, the increased transport efficiency caused by the E343D substitution suggests that a shorter side chain may allow metals to more rapidly reach the transport site due to a larger pore size of the selectivity filter and/or an expedited release from the transport site due to a longer distance between the bound metal and the carboxylic acid group and thus a weaker interaction. Using homology modeling, we generated the structural models of all 47 variants studied in this work in the outward-facing conformation (Fig. S4). Neither structural clashes nor shift of transmembrane helices were noticed in these models. Interestingly, the aspartate residue introduced at position 343 swings its side chain away from the other two residues in the selectivity filter in all the variants containing the E343D mutation. Due to the limitation of homology modeling, the dynamics of this residue is not available. Nevertheless, this result supports the notion that the E343D mutation likely increases the pore size. These effects on the structures of the selectivity filter and the transport site may also explain why the E343D variant becomes more promiscuous than wild-type ZIP8 (Fig. 5). The increased promiscuity makes this variant a better choice than the wild-type ZIP8 for later directed evolution study,^44,45^ and this variant may also be used in scenarios where a ZIP8 with an enhanced transport activity is preferred.

In contrast to the activity-enhancing E343D mutation, the D318E substitution drastically reduced the transporter activity across the entire spectrum of metal substrates. This may be attributed to a smaller pore size of the selectivity filter and a reduced volume of the transport site. However, the activity reduction associated with the D318E mutation could not be rescued by the E343D substitution, despite the fact that these two residues in the WT structure face each other in the selectivity filter and the transport site (Fig. 1). The lack of epistatic effects of contacting residues suggests that D318 and E343 have non-overlapping functions. In fact, the variants with the same amino acid composition at the selectivity filter but different distribution among the three key residues often did not show the same activity and substrate preference (Fig. S3). We conclude that it is not simply the amino acid composition but also the side chain positions in the selectivity filter and the transport site that are important for transport activity and substrate preference. Consistently, it was noticed that the glutamate residue introduced at position 318 induces a small displacement of E344, a residue in the transport site (Fig. S4), in all the variants containing the D318E mutations. Thus, the effect of this mutation may extend beyond its impact on the pore size of the selectivity filter.

Importantly, our results also reveal a remarkable potential of the ZIP fold to transport metal ions with diverse physicochemical properties. It is unexpected that Pb^2+^, a soft, non-d-block metal ion is able to translocate through ZIP8 (Fig. 2), because Pb^2+^ has an ionic radius 23% larger than Cd^2+^ (97 pm), the previously largest ZIP substrate, making ZIP8 as promiscuous as DMT1, which is known to transport a wide variety of divalent metal ions, including Pb^2+^.^46^ Some ABC transporters^47^ and p-type ATPases, such as ZntA,^48–50^ also transport Pb^2+^ but as a mean to expel this toxic metal from cells. Understanding the detailed Pb^2+^ transport processes by these metal transporters, which utilize distinct transport modes (elevator for ZIPs,^41^ rocking bundle for DMT1,^51^ and the ATP hydrolysis-driven alternating access for ABC transporters and p-type ATPases^52,53^) will provide critical insights into the mechanisms of cellular lead import and efflux. It was reported that lead induced ZIP8 expression in bovine aortic endothelial cells, but knocking down ZIP8 did not affect lead accumulation.^54^ Given that Pb^2+^ is not a preferred substrate (Fig. 4A) and Pb^2+^ transport can be largely suppressed by competing high-affinity metals, such as Zn^2+^ (Fig. 2B), ZIP8 may not be a primary transporter responsible for lead import. However, ZIP8 may still play a role in cellular lead uptake under certain circumstances. For instance, ZIP8, which is highly expressed in the lung,^55^ may be responsible for lead absorption through inhalation, a major pathway for lead exposure in humans, by transporting the dissolved lead particles in the alveolar fluid,^56^ as it possibly does for airborne manganese.^57^ Given that several organs with ZIP8 expression,^55^ such as the brain, kidney, liver, and spleen, are also susceptible to lead toxicity,^58^ the potential involvement of ZIP8 in lead distribution and toxicity warrants further studies using various cellular and animal models.

A recent study reported Cu^2+^ transport activities for several human ZIPs expressed in HEK293 cells, including ZIP8.^42^ In fact, we detected a marginal Cu^2+^ transport activity for the E343D variant (Fig. 5) but not for wild-type ZIP8 (Fig. 2). We noticed that there are differences in experimental settings, such as the absence of EDTA or other metal chelators in wash buffer used in that work, and these may account for some differences. We also found that VO^2+^ is not a substrate for wild-type ZIP8, but the new activity towards this diatomic ion was detected for the E343D variant (Fig. 5). The new transport activities for Pb^2+^, VO^2+^ and Cu^2+^, together with the highly variable activity and substrate preference observed in the variant library, demonstrate that the ZIP fold, which is uniquely present and conserved in the ZIP family,^41^ exhibits a remarkable plasticity and amenability, enabling it to transport a wide range of metal ions. This flexibility suggests that it could serve as a versatile scaffold for creating novel metal transporters with significantly expanded or tailored substrate specificity. For instance, the ZIP8 variants with increased substrate specificity toward one metal over others may be valuable tools to dissect the multi-functions of ZIP8 in physio-pathological scenarios involving different metals.^59–64^ The results from this work will also serve as a guide for changing the substrate specificity of the ZIPs from other species for applications in agriculture and environmental protection.

## Materials and methods

### Gene, plasmids, and reagents

The complementary DNA of human ZIP8 (GenBank access number: BC012125) from Mammalian Gene Collection were purchased from GE Healthcare. The ZIP8 construct consists of the N-terminal signal peptide of ZIP4 (amino acid residues 1–22) followed by a GSGS linker and a FLAG tag, and the ZIP8 coding sequence (residue 23–460). Site-directed mutagenesis of ZIP8 was conducted using QuikChange mutagenesis kit (Agilent, Cat# 600250). All mutations were verified by DNA sequencing. ^70^ZnO was purchased from American Elements (ZN-OX-01-ISO.070I, Lot# 1871511028-401) and ^57^FeCl_3_ was purchased from Sigma-Aldrich (790 427, Lot# MBBD4771). 30 mg of zinc oxide powder was dissolved in 5 mL of 1 M HCl and then diluted with ddH_2_O to make the stock solution at the concentration of 50 mM. ^57^FeCl_3_ was dissolved in 1 M HCl to final concentration of 100 mM. The ^70^Zn sample was certified as 72% abundance and ^57^Fe was certified as 99.9% abundance. Other reagents were purchased from Sigma-Aldrich or Fisher Scientific.

### Cell culture, transfection, and western blot

Human embryonic kidney cells (HEK293T, ATCC, Cat# CRL-3216) were cultured in Dulbecco's modified eagle medium (DMEM, Thermo Fisher Scientific, Invitrogen, Cat# 11965092) supplemented with 10% (v/v) fetal bovine serum (FBS, Thermo Fisher Scientific, Invitrogen, Cat# 10082147) and 1% antibiotic–antimycotic solution (Thermo Fisher Scientific, Invitrogen, Cat# 15240062) at 5% CO_2_ and 37 °C. Cells were seeded on polystyrene 24-well trays (Alkali Scientific, Cat#TPN1024) coated with poly-d-lysine (Corning, Cat# 354210) for 16 h and transfected with 0.8 μg DNA/well using lipofectamine 2000 (Thermo Fisher Scientific, Invitrogen, Cat# 11668019) in DMEM with 10% FBS.

For western blot, samples were mixed with the SDS sample loading buffer and heated at 37 °C for 20 min before loading on SDS-PAGE gel. The proteins were separated by SDS-PAGE and transferred to PVDF membranes (Millipore, Cat# PVH00010). After blocking with 5% (w/v) nonfat dry milk, the membranes were incubated with anti-FLAG antibody (Agilent, Cat# 200474-21) at 1 : 3000 or anti β-actin (Cell Signaling, Cat# 4970S) at 1 : 2500 at 4 °C overnight, which were detected with HRP-conjugated goat anti-rat immunoglobulin-G at 1 : 5000 dilution (Cell Signaling Technology, Cat# 7077S) or goat anti-rabbit immunoglobulin-G at 1 : 3000 dilution (Cell Signaling Technology, Cat# 7074S) respectively using the chemiluminescence reagent (VWR, Cat# RPN2232). The images of the blots were taken using a Bio-Rad ChemiDoc Imaging System.

### Metal transport assay

Twenty hours post transfection, cells were washed with a chelator-free, serum-free incubation buffer (10 mM Hepes, 142 mM NaCl, 5 mM KCl, 10 mM glucose, pH 7.3) followed by incubation with the incubation buffer plus metals for 30 min. To screen substrate and non-substrate metals for wild-type ZIP8 (Fig. 1), the metal mixture contained the following compounds: 2 mM CaCl_2_, 1 mM MgCl_2_, 5 μM for CdCl_2_, CoCl_2_, MnCl_2_, Pb(NO_3_)_2_, ^57^FeCl_3_, ^70^ZnCl_2_, VoCl_2_, NiCl_2_, CuCl_2_, AuCl_3_ (as [AuCl_4_]^−^), and PtCl_4_ (as [PtCl_6_]^2−^). Given the *K*_sp_ of PbCl_2_ (1.6 × 10^−5^ at 20 °C), Pb^2+^ at 5–50 μM in the above buffer is soluble. To screen variants against metal substrates, metal mixture set one is composed of 2 mM CaCl_2_, 1 mM MgCl_2_, 5 μM CdCl_2_, CoCl_2_, MnCl_2_, Pb(NO_3_)_2_, ^57^FeCl_3_, and ^70^ZnCl_2_ in the incubation buffer. Metal mixture set two is composed of 2 mM CaCl_2_, 1 mM MgCl_2_, 2 μM of CdCl_2_, 15 μM of MnCl_2_, 40 μM of ^57^FeCl_3_ and 1 mM of ascorbic acid in the incubation buffer. After incubation at 37 °C for 30 min, the 24-well plates were put on ice and an equal volume of the ice-cold washing buffer containing 1 mM EDTA was added to the cells to terminate metal uptake, followed by three times of washing with ice-cold wash buffer before cell lysis in nitric acid.

For kinetic studies, cells were grown and transfected in the same way as in the metal transport assay. Briefly, twenty hours post transfection, cells were washed with the same incubation buffer, followed by incubation with the buffer plus metals for 30 min. All metal solutions contained 2 mM CaCl_2_ and 1 mM MgCl_2_, with different concentrations of metals as indicated. 1 mM ascorbic acid was included when the transport assay for Fe^2+^ was conducted. After incubation at 37 °C for 30 minutes, 24-well plates were put on ice and an equal volume of the ice-cold washing buffer containing 1 mM EDTA was added to cells to terminate metal uptake, followed by three times of washing with ice-cold wash buffer before cell lysis in nitric acid.

### ICP-MS experiment

The ICP-MS experiments were conducted as described.^31^ All standards, blanks, and cell samples were prepared using trace metal grade nitric acid (70%, Fisher chemical, Cat# A509P212), ultrapure water (18.2 MΩ cm @ 25 °C), and metal free polypropylene conical tubes (15 and 50 mL, Labcon, Petaluma, CA, USA). For cell samples in polystyrene 24-well cell culture plates, 200 μl of 70% trace nitric acid was added to allow for initial sample digestion. Following digestion, 150 μl of the digested product was transferred into metal free 15 mL conical tubes. For liquid samples, 50 μl of liquid samples were added to metal free conical tubes followed by addition of 150 μl of 70% trace nitric acid. All cell and liquid samples were then incubated at 65 °C in a water bath for one hour followed by dilution to 5 mL using ultrapure water. The completed ICP-MS samples were analyzed using an Agilent 8900 Triple Quadrupole ICP-MS (Agilent, Santa Clara, CA, USA) equipped with the Agilent SPS 4 Autosampler, integrate sample introduction system (ISiS), x-lens, and micromist nebulizer. Daily tuning of the instrument was accomplished using manufacturer supplied tuning solution containing Li, Co, Y, Ce, and Tl. Global tune optimization was based on optimizing intensities for ^7^Li, ^89^Y, and ^205^Tl while minimizing oxides (^140^Ce^16^O/^140^Ce < 1.5%) and doubly charged species (^140^Ce++/^140^Ce+ < 2%). Following global instrument tuning, gas mode tuning was accomplished using the same manufacturer supplied tuning solution in KED mode (using 100% UHP He, Airgas). Specifically, intensities for ^59^Co, ^89^Y, and ^205^Tl were maximized while minimizing oxides (^140^Ce^16^O/^140^Ce < 0.5%) and doubly charged species (^140^Ce++/^140^Ce+ < 1.5%) with short term RSDs <3.5%. ICP-MS standards were prepared from a stock solution of NWU-16 multi-element standard (Inorganic Ventures, Christiansburg, VA, USA) that contains the metals studied in this work that were diluted with 3% (v/v) trace nitric acid in ultrapure water to a final element concentration of 1000, 500, 250, 125, 62.5, 31.25, and 0 (blank) ng g^−1^ standard. Internal standardization was accomplished inline using the ISIS valve and a 200 ng g^−1^ internal standard solution in 3% (v/v) trace nitric acid in ultrapure water consisting of Bi, In, ^6^Li, Sc, Tb, and Y (IV-ICPMS-71D, Inorganic Ventures, Christiansburg, VA, USA). ^6^Li, ^45^Sc, and ^89^Y were used for internal standardization. Continuing calibration blanks (CCBs) were run every 10 samples and a continuing calibration verification standard was analyzed at the end of every run for a 90–110% recovery.

### Flow cytometry experiment

To detect the total expression level, the cells were fixed with 4% formaldehyde at room temperature for 15 min, blocked and permeabilized for 1 h with Dulbecco's phosphate-buffered saline (DPBS) containing 2% BSA and 0.1% Triton X-100, and then incubated with anti-FLAG antibody (Sigma, F3165) at 1 : 500 in 2% BSA in PBST containing 0.1% Tween-20 (ThermoFisher Scientific, Cat# 85113) for 2 hours on ice. After the unbound antibodies were removed by washing with DPBS, the cells were incubated with an AlexaFluor 568-conjugated goat anti-mouse antibody (Invitrogen, CAT# A-11004) at 1 : 1000 diluted in DPBS with 2% BSA for 1 h. After washing three times with a FACS buffer (2% BSA, 1 mM EDTA, 20 mM HEPES in DPBS without Ca^2+^ and Mg^2+^), the cells were resuspended by FACS buffer for flow cytometry analysis. To detect the surface expression level, the procedure was the same except that Triton X-100 and Tween-20 were excluded. The cells were applied to a ThermoFisher Attune Cytpix flow cytometer. 10 000 cells were assessed for each condition. The single cells were selected (Fig. S5) and then analysed in the YL-1 channel to detect the fluorescence of AlexaFluor-568. The mean fluorescence intensity (MFI) of each population was calculated and the histogram charts were generated to compare the signals from each group (Fig. S5). All the data processing was performed in FlowJo 10.8.1 and GraphPad Prism 10.

### Structural modelling

To generate the structural model of wild-type ZIP8 in the outward-facing conformation shown in Fig. 1, we first retrieved the structural model of human ZIP13 (Uniprot ID Q96H72) from the AlphaFold protein structure database (https://alphafold.com/entry/Q96H72). In this model, the protein is in an outward-facing conformation. Using this model as the template, the structural model of human ZIP8 was generated using homology modelling by SWISS MODEL (https://swissmodel.expasy.org/). Similarly, homology modelling was conducted to generate the structural models of the variants studied in this work, and the results are summarized in Fig. S4.

## Statistics

We assumed a normal distribution of the samples and significant difference were examined using two-tail Student's *t*-test. Uncertainties shown in figures are reported as standard deviation (S.D.) or standard error (S.E).

## Author contributions

J. H., Y. J., M. N., T. W., K. M., and T. V. O. conceived the project and designed the experiments, Y. J., M. N., T. W., and K. M. conducted the experiments; Y. J., M. N., T. W., K. M., T. V. O., and J. H. analyzed the data and wrote the manuscript.

## Conflicts of interest

The authors declare that they have no conflicts of interest with the contents of this article.

## Supplementary Material

SC-OLF-D5SC03700J-s001

## Data Availability

Supplementary information: All raw and processed data reported in the main text and SI are available upon request. See DOI: https://doi.org/10.1039/d5sc03700j.
