# Supplementary material for: Targeting the selectivity filter to drastically alter the activity and substrate spectrum of a promiscuous metal transporter
Source: Chem Sci. 2025 Aug 26;16(38):17675–87. doi: 10.1039/d5sc03700j (PMC12406047; doi:10.1039/d5sc03700j)
Supplement: SC-016-D5SC03700J-s001 [file SC-016-D5SC03700J-s001.pdf]

Supplementary Information (SI) for Chemical Science.
This journal is © The Royal Society of Chemistry 2025

Supplementary Information

Targeting the selectivity filter to drastically alter the activity and substrate spectrum of a promiscuous metal transporter

Yuhan Jiang,[1,*] Michael Nikolovski,[2,*] Tianqi Wang,[2] Keith MacRenaris,[1,3,4,5] Thomas V. O'Halloran,[1,3,4,5,‡] Jian Hu[1,2,‡]

[1]Department of Chemistry, Michigan State University, East Lansing, Michigan, USA

[2]Department of Biochemistry and Molecular Biology, Michigan State University, East Lansing, Michigan, USA

[3]Department of Microbiology, Genetics, & Immunology, Michigan State University, East Lansing, Michigan, USA

[4]Elemental Health Institute, Michigan State University, East Lansing, Michigan, USA

[5]Quantitative Bio Element Analysis and Mapping (QBEAM) Center, Michigan State University, East Lansing, Michigan, USA

*Equally contributive to this work
‡Corresponding authors: Thomas V. O'Halloran (ohallor8@msu.edu); Jian Hu (hujian1@msu.edu)

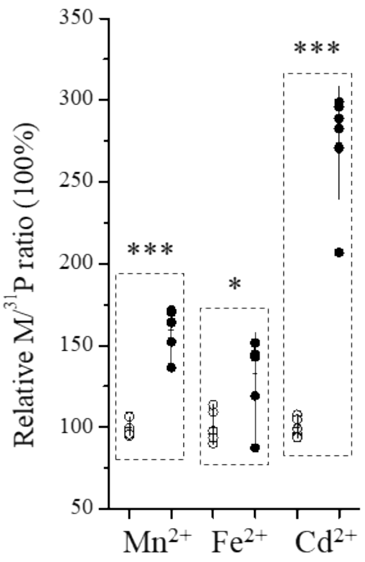

Figure S1. Detection of the activities for Mn^{2+}, Fe^{2+}, and Cd^{2+} under the optimized condition. For each metal, the relative M/^{31}P ratio of the ZIP8 group (solid circle) is expressed as a percentage of the M/^{31}P ratio of the empty vector group (open circle). Each data point represents the result of one sample, and six replicates were conducted under one condition. For the result of each metal, the horizontal bar is the mean of three replicates and the vertical bar shows 1±S.D. Two tailed student's *t* test was used to test statistical significance. *: P<0.05; ***: P<0.001. The P values for Mn^{2+}, Fe^{2+}, and Cd^{2+}, are $1x10^{-6}$, 0.01, and $2x10^{-7}$, respectively.

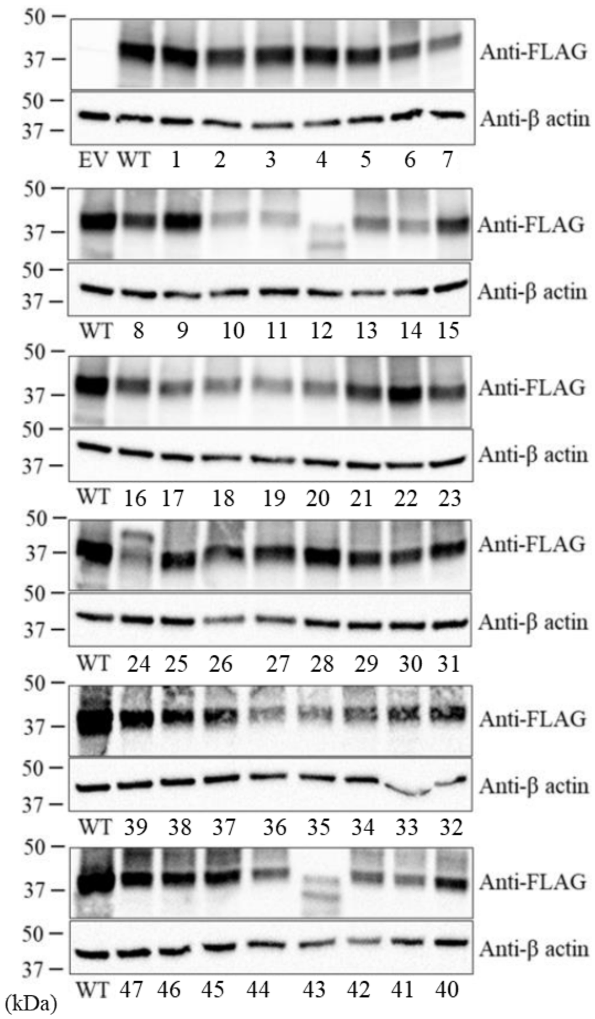

Variant#	Substitutions
1	Q180D
2	Q180E
3	Q180H
4	Q180N
5	Q180S
6	D318E
7	E343D
8	E343H
9	E343Q
10	Q180D/D318E
11	Q180E/D318E
12	Q180H/D318E
13	Q180N/D318E
14	Q180S/D318E
15	Q180D/E343D
16	Q180E/E343D
17	Q180H/E343D
18	Q180N/E343D
19	Q180S/E343D
20	Q180D/E343Q
21	Q180E/E343Q
22	Q180H/E343Q
23	Q180N/E343Q
24	Q180S/E343Q
25	D318E/E343D
26	D318E/E343Q
27	Q180D/E343H
28	Q180E/E343H
29	Q180H/E343H
30	Q180N/E343H
31	Q180S/E343H
32	D318E/E343H
33	Q180D/D318E/E343D
34	Q180D/D318E/E343Q
35	Q180E/D318E/E343D
36	Q180E/D318E/E343Q
37	Q180N/D318E/E343D
38	Q180N/D318E/E343Q
39	Q180S/D318E/E343D
40	Q180S/D318E/E343Q
41	Q180D/D318E/E343H
42	Q180E/D318E/E343H
43	Q180H/D318E/E343D
44	Q180H/D318E/E343H
45	Q180H/D318E/E343Q
46	Q180N/D318E/E343H
47	Q180S/D318E/E343H

Figure S2. Expression of N-terminal FLAG-tagged ZIP8 and its variants detected by Western blot. An anti-FLAG antibody was used to detect the total expression of ZIP8 and its variants. β-actin was used as the loading control. Variants #12 (Q180H/D318E), #24 (Q180S/E343Q), and #43 (Q180H/D318E/E343D) showed different molecular weights from other constructs and exhibited poor activities (**Table 1**).

Construct	Amino Acid			Metal Mixture_Set 1				Metal Mixture_Set 2	
	180	318	343	Co	Zn	Cd	Pb	Mn	Cd
Q180E/E343D	E	D	D	179	211	158	152	64	193
Q180D	D	D	E	24	119	81	125	45	104
Q180D/D318E/E343D	D	E	D	16	27	35	44	56	34
Q180H/E343D	H	D	D	111	268	182	84	131	189
Q180D/E343H	D	D	H	69	196	129	195	53	153
E343D	Q	D	D	718	223	370	427	482	150
Q180D/E343Q	D	D	Q	191	441	296	413	94	157
Q180H	H	D	E	33	168	105	34	73	138
Q180E/E343H	E	D	H	166	232	158	165	45	185
Q180D/D318E/E343H	D	E	H	11	31	39	58	52	29
Q180H/D318E/E343D	H	E	D	9	10	6	0	8	3
WT	Q	D	E	100	100	100	100	100	100
Q180E/E343Q	E	D	Q	224	538	477	593	161	217
318E/E343D	Q	E	D	0	35	41	38	45	35
Q180D/D318E/E343Q	D	E	Q	19	30	29	57	57	29
Q180H/D318E	H	E	E	13	16	25	63	34	11
Q180E/D318E/E343H	E	E	H	17	36	36	172	41	26
Q180H/D318E/E343Q	H	E	Q	18	85	45	0	38	29
318E/E343H	Q	E	H	0	9	16	46	25	21
Q180H/E343Q	H	D	Q	307	491	399	194	122	253
E343H	Q	D	H	48	73	135	71	33	26
Q180E/D318E/E343D	E	E	D	16	38	41	57	58	36
Q180D/D318E	D	E	E	0	25	37	16	42	21
Q180E	E	D	E	34	131	82	94	54	109
Q180E/D318E/E343Q	E	E	Q	7	40	37	61	49	28
D318E	Q	E	E	18	31	40	125	58	17

Figure S3. Comparison of the constructs with the same amino acid composition at the selectivity filter. The constructs in the same frame have the same amino acid composition but different distribution at the positions of 180, 318, and 343. They often do not exhibit the same activity or substrate preference, suggestive of non-overlapping roles of these residues in transport. Data are retrieved from **Table 1**.

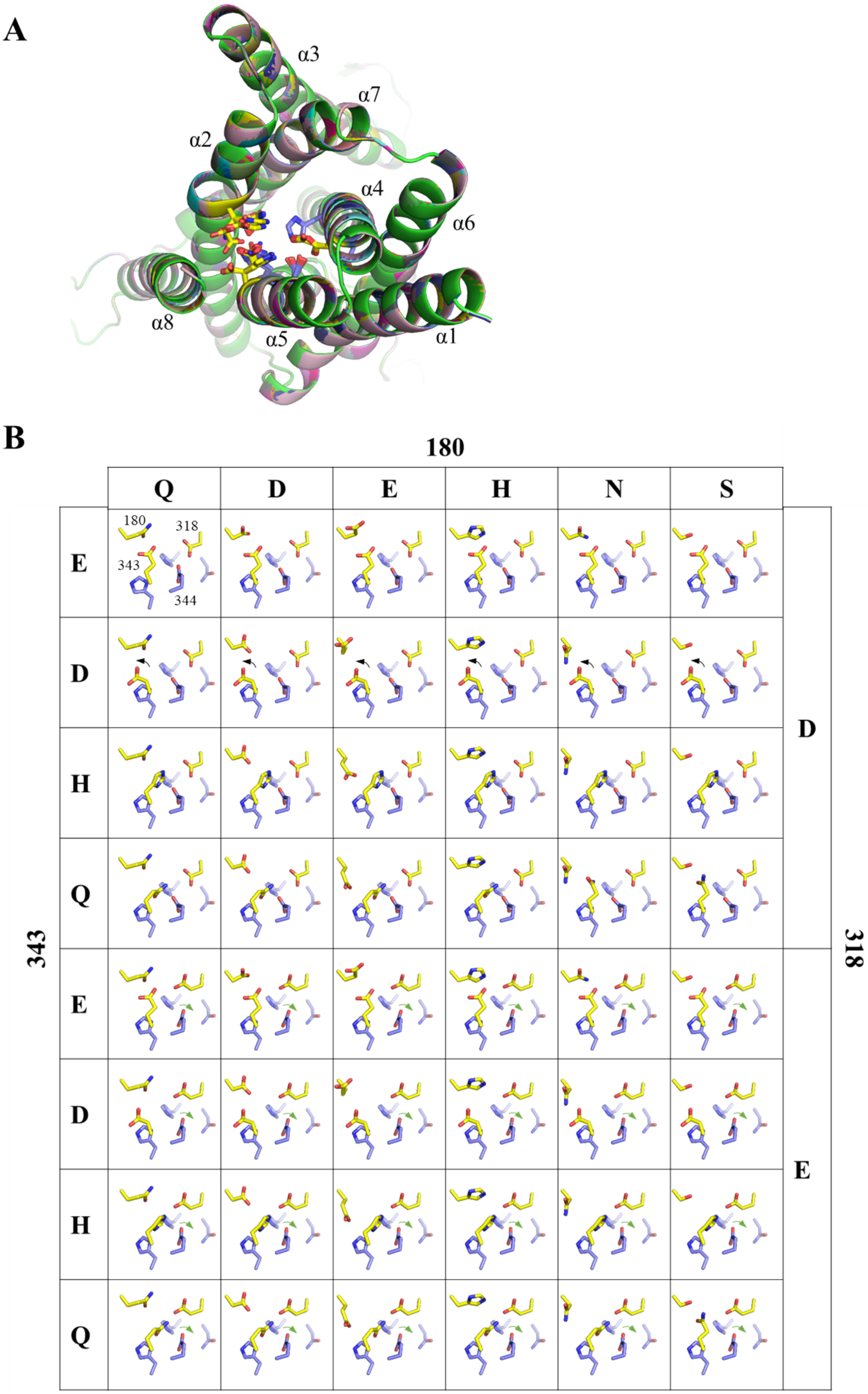

A
α3
α7
α2
α4
α6
α8
α5
α1
B
180
Q
D
E
H
N
S
343
E
D
H
Q
E
D
H
Q
318
D
E
180
318
343
344

Figure S4. Structural comparison of the selectivity filters of the ZIP8 variants with the wild type protein. (**A**) Superimposed 48 structural models generated by homology modeling. The structures are shown in top view. The transmembrane helices are labeled from α1 to α8. The residues forming the selectivity filter are in yellow and the residues forming the transport site are in blue. (**B**) Comparison of the selectivity filters and transport sites of the 48 constructs. The wild type ZIP8 is shown in the upper left corner. The black arrows in the second row indicate the swing of the aspartate side chain at position 343, and the variants containing the E343D mutation showed increased activities for all metal substrates. In contrast, the green arrows in the last four rows indicate the shift of the side chain of E344 in the variants containing the D318E mutation. These variants showed largely diminished transport activities for nearly all metal substrates.

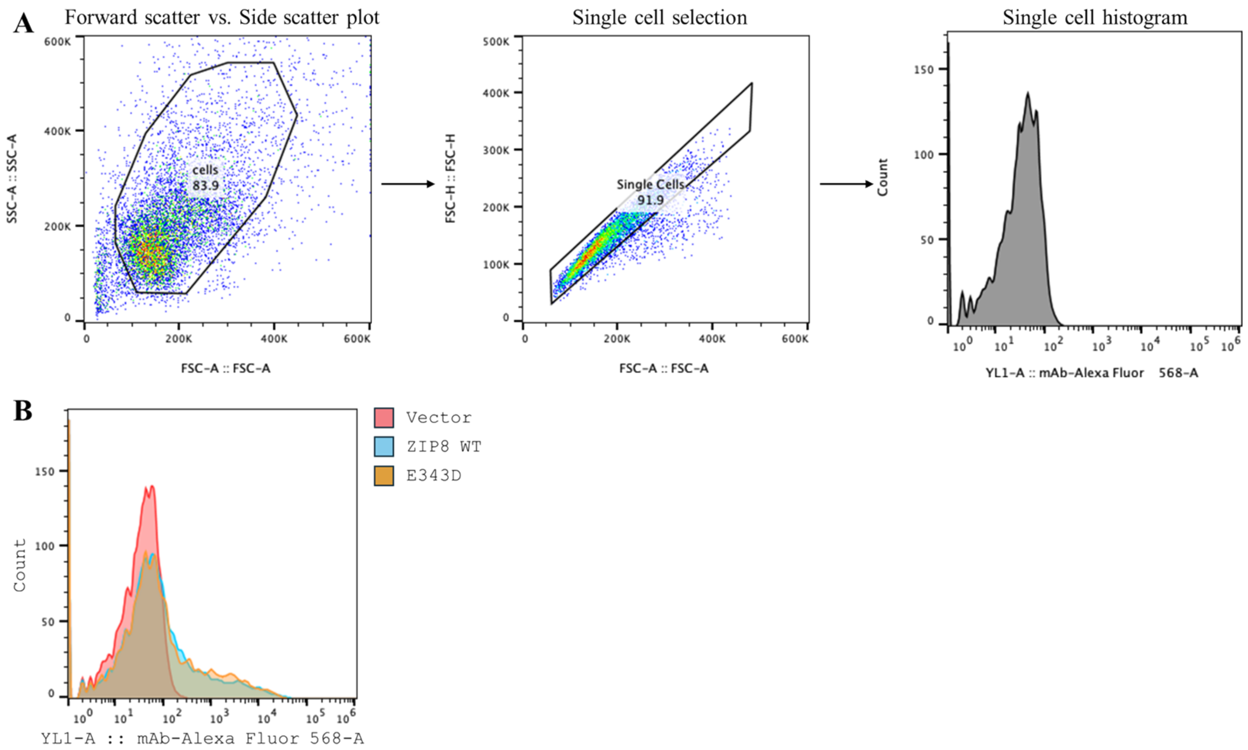

Figure S5. Flow cytometry data processing. (**A**) Representative data processing. (**B**) Comparison of cell surface expression levels of empty vector, ZIP8, and the E343D variant in a histogram chart.
